# Ketogenic diet induces an inflammatory reactive astrocytes phenotype reducing glioma growth

**DOI:** 10.1007/s00018-025-05600-4

**Published:** 2025-02-08

**Authors:** Maria Rosito, Javeria Maqbool, Alice Reccagni, Micol Mangano, Tiziano D’Andrea, Arianna Rinaldi, Giovanna Peruzzi, Beatrice Silvestri, Alessandro Rosa, Flavia Trettel, Giuseppina D’Alessandro, Myriam Catalano, Sergio Fucile, Cristina Limatola

**Affiliations:** 1 Department of Physiology and Pharmacology, Sapienza University, P.Le Aldo Moro 5, 00185 Rome, Italy; 2 Center for Life Nanoscience & Neuroscience, Istituto Italiano di Tecnologia@Sapienza, Rome, Italy; 3 IRCCS Neuromed, Pozzilli, IS Italy; 4 Department of Biology and Biotechnologies “Charles Darwin”, Sapienza University of Rome, Rome, Italy; 5 Department of Physiology and Pharmacology, Laboratory Affiliated to Institute Pasteur Italia, Sapienza University, P.Le Aldo Moro 5, 00185 Rome, Italy

**Keywords:** Astrocytes, Ketogenic diet, Microglia, Glioma, β-HB, Astrogliosis, Pro-inflammatory astrocytes

## Abstract

The use of a ketogenic diet (KD) in glioma is currently tested as an adjuvant treatment in standard chemotherapy regimens. The metabolic shift induced by the KD leads to the generation of ketone bodies that can influence glioma cells and the surrounding microenvironment, but the mechanisms have not yet been fully elucidated. Here, we investigated the potential involvement of glial cells as mediators of the KD-induced effects on tumor growth and survival rate in glioma-bearing mice. Specifically, we describe that exposing glioma-bearing mice to a KD or to β-hydroxybutyrate (β-HB), one of the main KD metabolic products, reduced glioma growth in vivo, induced a pro-inflammatory phenotype in astrocytes and increased functional glutamate transporters. Moreover, we described increased intracellular basal Ca^2+^ levels in GL261 glioma cells treated with β-HB or co-cultured with astrocytes. These data suggest that pro-inflammatory astrocytes triggered by β-HB can be beneficial in counteracting glioma proliferation and neuronal excitotoxicity, thus protecting brain parenchyma.

**Supplementary Information:**

The online version contains supplementary material available at 10.1007/s00018-025-05600-4.

## Introduction

In recent years, the elements in favor of the use of the ketogenic diet (KD) as a complementary approach to standard glioma therapy have grown. Preclinical and clinical evidences suggests that the KD may exert therapeutic benefits in glioma, by targeting metabolic vulnerabilities inherent to tumor cells while potentially enhancing the efficacy of standard treatments [1–3].

The metabolic shift induced by the KD leads to the generation of ketone bodies, particularly β-hydroxybutyrate (β-HB), that can be used as an alternative energy source. Interestingly, while normal brain cells, including neurons and glia, readily metabolize ketone bodies [4], it has been hypothesized that glioma cells are not able to utilize ketone bodies for energy production [5]. This metabolic discrepancy between tumor cells and normal brain cells provides a potential therapeutic window for exploiting the KD in glioma treatment. Recent studies highlighted the interplay between metabolic reprogramming in tumor cells and the surrounding microenvironment, suggesting that adjuvant treatments such as a controlled KD may also modulate the activity of microglia and astrocytes, potentially influencing glioma progression [6].

Studies have shown that the KD leads to a shift in astrocyte metabolism towards increased utilization of ketone bodies, such as β-HB, as an alternative to glucose as energy source [7]. This metabolic adaptation enhances astrocytic energy production improving mitochondrial performance in glia and neurons [8] thereby promoting neuronal survival and function under conditions of metabolic stress [9]. In addition, emerging evidence suggests that the KD modulates microglial metabolism, leading to alterations in their phenotype and function; specifically, the KD has been shown to reduce microglial activation and pro-inflammatory cytokines production, while enhancing their phagocytic activity and neuroprotective properties [10].

Additionally, beyond its metabolic effects, emerging evidence suggests that β-HB is a signaling metabolite [11] and a powerful epigenetic molecule in the brain through direct and specific histone marks remodeling [12, 13]. It has been reported that in response to the KD, microglia may adopt an anti-inflammatory phenotype [14], which in principle could contribute to their pro-tumoral effect [15]. However, in the context of glioma, our understanding of the phenotypic changes induced by the KD in glial cells remains almost unexplored.

Here, we investigated the effect of the KD and β-HB in glioma-bearing mice showing a reduction in tumor growth and an increased median survival rate respect to mice fed with a matched control diet (CD). To explore a possible mechanism by which KD might exert its function, we performed co-culture experiments showing that glial cells act as mediators of the KD effect on tumor growth.

Specifically, we describe that GL261 glioma cells are not able to use β-HB as an energetic fuel, and their proliferation rate slowed down by the presence of the pro-inflammatory astrocytes induced by β-HB treatment. In addition, we showed that pro-inflammatory astrocytes isolated from the glioma-bearing mice or induced by the β-HB treatment, exhibit increased expression of glutamate transporters and are functionally active in reducing the extracellular glutamate level. Moreover, we described increased intracellular basal Ca^2+^ levels in GL261 treated with β-HB or co-cultured with astrocytes. All these data suggest that β-HB, triggering a pro-inflammatory astrocytes phenotype, can reduce glioma proliferation, counteract excitotoxicity, and dysregulate Ca^2+^ homeostasis of glioma cells thus proving a beneficial effect on brain parenchyma.

## Results

### The restricted ketogenic diet in glioma bearing-mice reduces tumor volume and improves mice survival

**Fig. 1:**
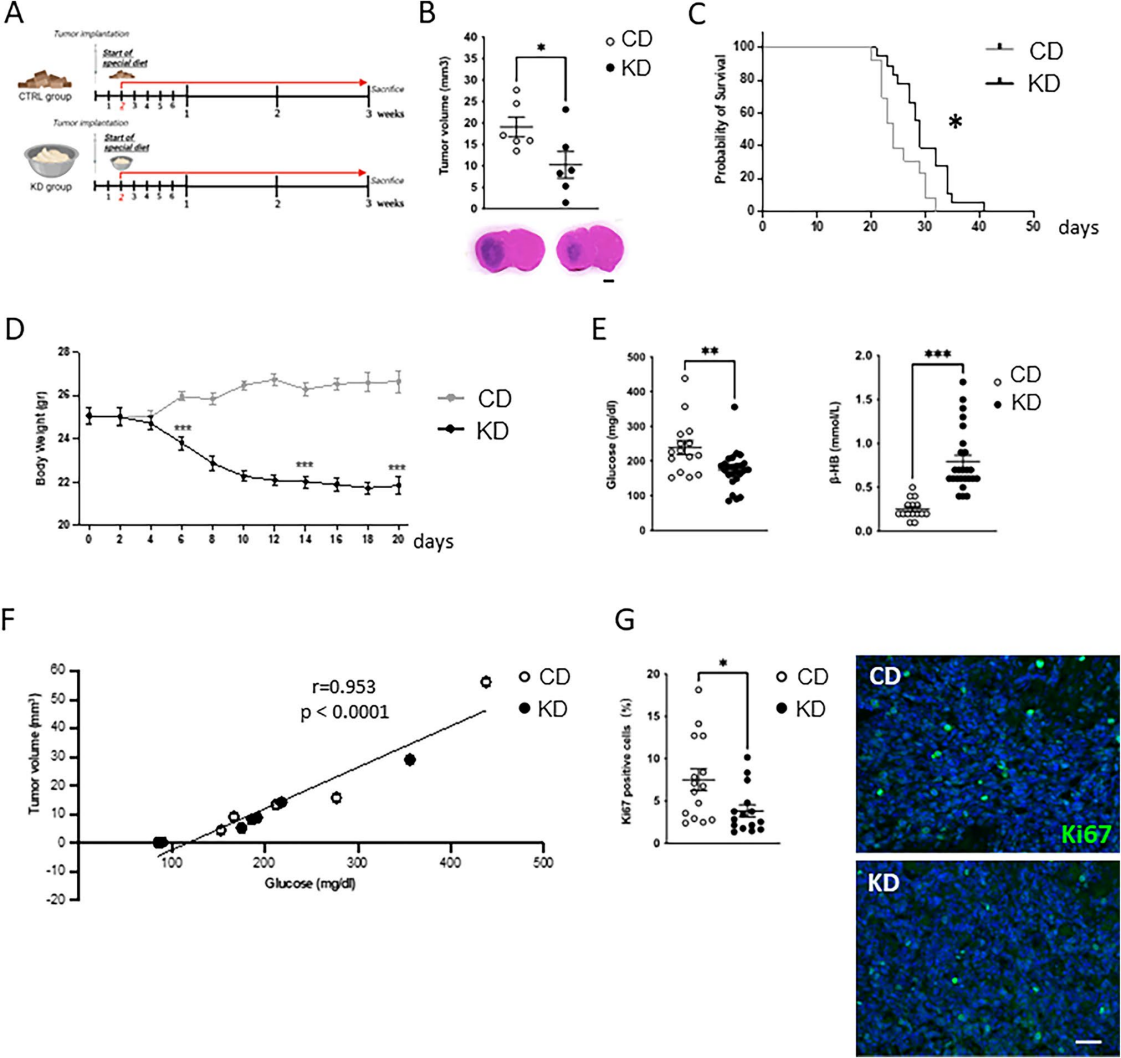
Effect of the KD on tumor growth and mice survival. **A** Study design. **B** Tumor size in CD and KD mice, *n* = 6, pooled from two experiments, three animals per group. Data are presented as the mean ± SEM *p < 0.05, Student’s t-test. Right pictures: Representative images of brain coronal slices, scale bar = 1 mm. **C** Kaplan–Meier survival curves of CD and KD GL261 bearing mice, *p = 0.012, Log-rank test. **D** Body weight measurements of CD and KD GL261 bearing mice. Data are presented as the mean ± SEM ***p < 0.001 Student’s t-test evaluated on days 6, 14, and 20. **E** Dot plot showing the blood glucose and β-HB level of CD and KD mice measured at day 14 after tumor injection. Data are presented as the mean ± SEM **p < 0.005, ***p < 0.001 Student’s t-test. **F** Dot plot showing the correlation between glucose levels and tumor volume in CD (n = 5) and KD (n = 7) mice. r = 0.953; p < 0.0001. **G** Dot plot showing the percentage of Ki67 positive cells in the tumor core area (CD: 15/3 slices/mice; KD: 15/3 slices/mice) Data are presented as the mean ± SEM *p < 0.05, Student’s t-test. (Left) Representative images of Ki67 (green) in CD and KD tumor core area. Hoechst staining (blu) for nuclei visualization; scale bar: 50 μm

Here we investigated the effect of a restricted and unrestricted ketogenic and the relative matched control diet (CD) on glioma-bearing mice (Figs. 1A, S1).

Three weeks after tumor injection, a reduction in tumor volume was observed only in mice fed with a restricted dietary regimen (KD: 14.88 ± 3.8, CD: 25.62 ± 6.5 mm^3^ *p = 0.031) (Fig. 1B), as well as an increase in mice survival (KD: 29, CD: 24 median survival days) (Fig. 1C). We also investigated the effect of an unrestricted KD, showing an increase in tumor volume in KD versus CD mice (KD: 15.8 ± 1.5, CD: 8.3 ± 2.3 mm^3^ standard diet: SD: 9.4 ± 2.5 mm^3^ *p = 0.04) (Fig. S1A).

During the different dietary regimens, the mice's body weight was constantly monitored showing a significant reduction in KD versus control diet (CD)-treatedmice starting 6 days after tumor implantation. The body weight reduction in restricted KD mice is constant during the experimental timeline and never falls below 12–13% of the original weight (Fig. 1D). At difference, the body weight of the unrestricted KD mice increased compared to CD and SD mice (Fig. S1A).

Given that the restricted ketogenic diet was beneficial in reducing tumor volumes, we employed this dietary regimen to further investigate the effectiveness in reducing tumor progression.

Since it has been shown that KD induces changes in the metabolic state [16], we evaluated the glucose and β-HB blood levels. At day 14 after tumor implantation, KD mice exhibit a reduction in plasma glucose (Fig. 1E, left) and an increase in plasma β-HB level (Fig. 1E, right) to CD mice.

Since we found that glucose levels are increased in glioma-bearing mice (239 ± 20.3 mg/dl n = 15 mice) in comparison with mice before tumor implantation (153.5 ± 9.6 mg/dl n = 12 mice) we tested the possible correlation with the tumor volume and found that the glycemic values observed at 14 days after tumor implantation are significantly positively associated (r = 0.952 and p < 0.0001) with the tumor volume (Fig. 1F). These data show that mice with a higher blood glucose level (fed either with CD or KD) exhibit a bigger tumor volume when compared to mice with a lower glucose level, indicating a positive relation between glycemia and tumor growth.

By immunofluorescence staining, we tested the proliferation of GL261 cells: the expression of Ki67 marker is reduced in the tumor core of KD mice compared to CD mice (KD: 3.85 ± 0.7 CD: 7.53 ± 1.26) (Fig. 1G). Altogether, these data confirm that KD is effective in reducing glioma growth and increasing mice survival.

### Glioma growth is not directly affected by β-HB

**Fig. 2:**
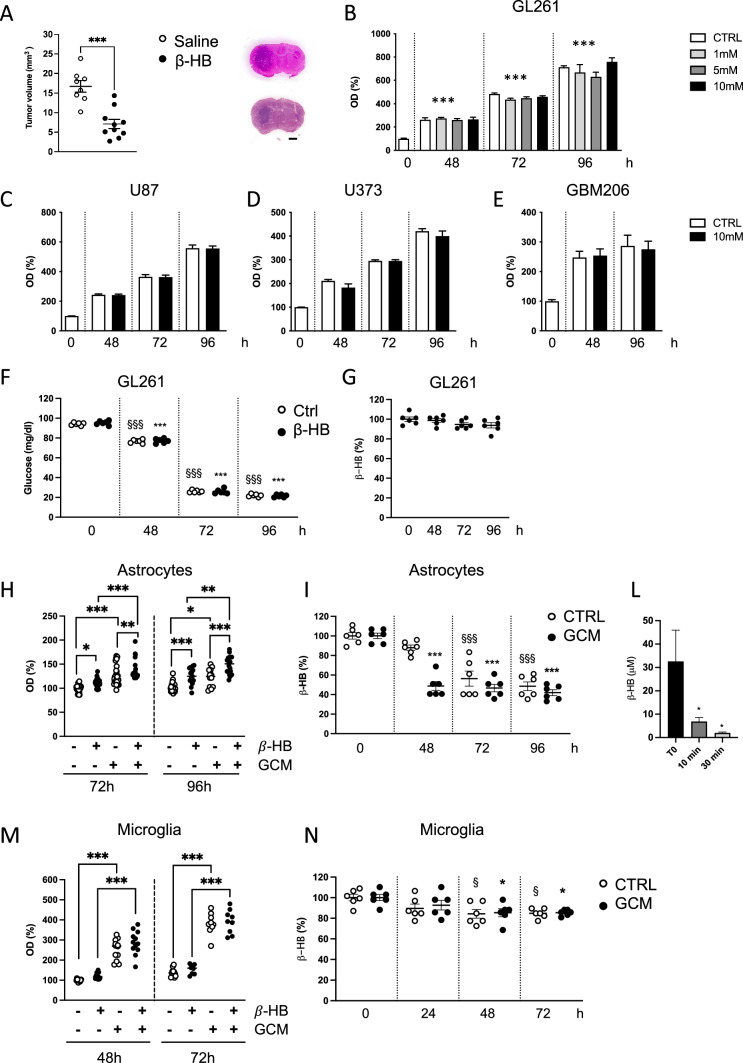
Effect of β-HB treatment on GL261 and astrocytes. **A** Tumor size in saline and β-HB injected mice, *n* = 8–10 animals per group. Data are presented as the mean ± SEM ***p < 0.001, Student’s t-test. Right pictures: Representative images of brain coronal slices, scale bar = 1 mm.** B** Bar plot showing the MTT assay on GL261 cells stimulated with different β-HB concentrations. Data are presented as the mean ± SEM *** p < 0.001. One-way ANOVA—Dunnett’s multiple comparison test versus time 0. **C** Bar plot showing the MTT assay on human glioma cells U87, on** D** U373 and **E** GBM206 primary cells from patient. Data are presented as the mean ± SEM ^§§§^ p < 0.001;*** p < 0.001. One-way ANOVA—Dunnett’s multiple comparison test versus its respective time 0. **F** Glucose consumption by GL261 cells without (white dots) or in the presence of β-HB (black dots). Data are presented as the mean ± SEM *** p < 0.001. One-way ANOVA—Dunnett’s multiple comparison test. **G** β-HB consumption by GL261 cells. Data are presented as the mean ± SEM. **H** MTT assay on astrocytes cultured with or without GCM and β-HB for 72 and 96 h of treatments. Data are presented as the mean ± SEM * p < 0.05 ** p < 0.005 *** p < 0.001 Two-way ANOVA. Fisher’s LSD test. **I** β-HB consumption by astrocytes: measurement of the normal extracellular medium (CTRL, white dots) or in the presence of GCM (black dots). Data are presented as the mean ± SEM ^§§§^ p < 0.001; *** p < 0.001. One-way ANOVA—Dunnett’s multiple comparison test versus its respective time 0. **L** Intracellular β-HB consumption by astrocytes measured at the indicated time point. Data are presented as the mean ± SEM * p < 0.05. One-way ANOVA—Dunnett’s multiple comparison test. **M** MTT assay on microglia cultured with or without GCM and β-HB for 48 and 72 h of treatments. Data are presented as the mean ± SEM *** p < 0.001 Two-way ANOVA. Fisher’s LSD test. **N** β-HB consumption by microglia without (CTRL, white dots) or in the presence of GCM (black dots). Data are presented as the mean ± SEM. ^§^ p < 0.05; * p < 0.05. One-way ANOVA—Dunnett’s multiple comparison test versus its respective time 0

Since KD results in β-HB production that can be oxidized as a brain fuel [17], we first tested the effect of this ketone body on glioma growth. We found that the intraperitoneal β-HB administration to mice is able to reduce tumor volume thus mimicking the effect of the ketogenic diet (β-HB: 7.12 ± 1.18, saline: 16.72 ± 1.5 mm^3^) (Fig. 2A). To investigate the mechanisms involved in the β-HB-induced reduction of glioma growth, we tested its effects on glioma and non-neoplastic glial cells. We first report that in vitro, β-HB does not change the GL261 (Fig. 2B) viability since the proliferation rate during the in vitro culture is unaffected. Similar results were obtained on a different murine glioma cell line, CT2A (Fig. S2A). We also tested the β-HB effect on two human GBM cells lines U87 (Fig. 2C) and U373 (Fig. 2D) and on primary GBM cells (GBM206) (Fig. 2E). The MTT assay confirmed that β-HB does not modulate the viability of all the tested GBM human cells. We measured the glucose and β-HB consumption of glioma cells in culture: while the glucose level is significantly reduced respect to the initial (time 0) concentration, without any differences between ctrl and β-HB treatment (Fig. 2F), the β-HB level remains constant (Fig. 2G). In addition, the transcriptional level of monocarboxylate transporters (Fig. S2B) and of β-HB converting enzymes (Fig. S2C), evaluated at 72 h of β-HB treatment, are not modulated, indicating that GL261 cells are not using or converting β-HB to generate energy.

In the hypothesis that the effect observed in vivo could be mediated by non-tumor cells, we focused on astrocytes because these cells have important roles in shaping the glioma microenvironment [18], but their role during ketosis is still unexplored. We investigated the effect of β-HB on astrocyte proliferation and found that upon 72 and 96 h of treatment, astrocytes exhibit an increased proliferation that is further enhanced in the presence of glioma-conditioned medium (GCM) (Fig. 2H). Parallel to this increased astrocytic proliferation rate, we measured a significant reduction of the extracellular β-HB level at 72 and 96 h in astrocyte cultures, already appreciable at 48 h when astrocytes were exposed to GCM (Fig. 2I). To analyze the ability of astrocytes to effectively consume β-HB, we exposed astrocytes to β-HB and analyzed the level of the intracellular ketone body by an enzymatic assay. The astrocytes are efficiently metabolizing the β-HB since its level is highly reduced already after 10 min (T10 = 6.86 ± 1.75 µM) compared to the level measured immediately after the treatment (T0 = 32.58 ± 13.41 µM). These levels are further reduced 30 min after the treatment (T30 = 1.99 ± 0.41 µM). In contrast, in GL261 cells we did not detect β-HB at the intracellular level.

The transcriptional levels of monocarboxylate transporters (Fig. S2D) and β-HB converting enzymes (Fig. S2E), evaluated at 72 h of β-HB treatment, are not modulated.

We also tested the β-HB effect on microglia proliferation, appreciating a significant increase in proliferation only in the presence of GCM at 48 and 72 h (Fig. 2L). In addition, a reduction of β-HB in the microglia culturing medium (Fig. 2M), is paralleled by the increased transcriptional level of monocarboxylate transporters (Fig. S2F) and β-HB converting enzymes (Fig. S2G).

These data suggest that GCM induced an increased proliferation of astrocytes and microglia and that β-HB treatment further increased the proliferation rate of astrocytes.

### Astrocytes acquired a pro-inflammatory phenotype upon KD and β-HB administration

**Fig. 3:**
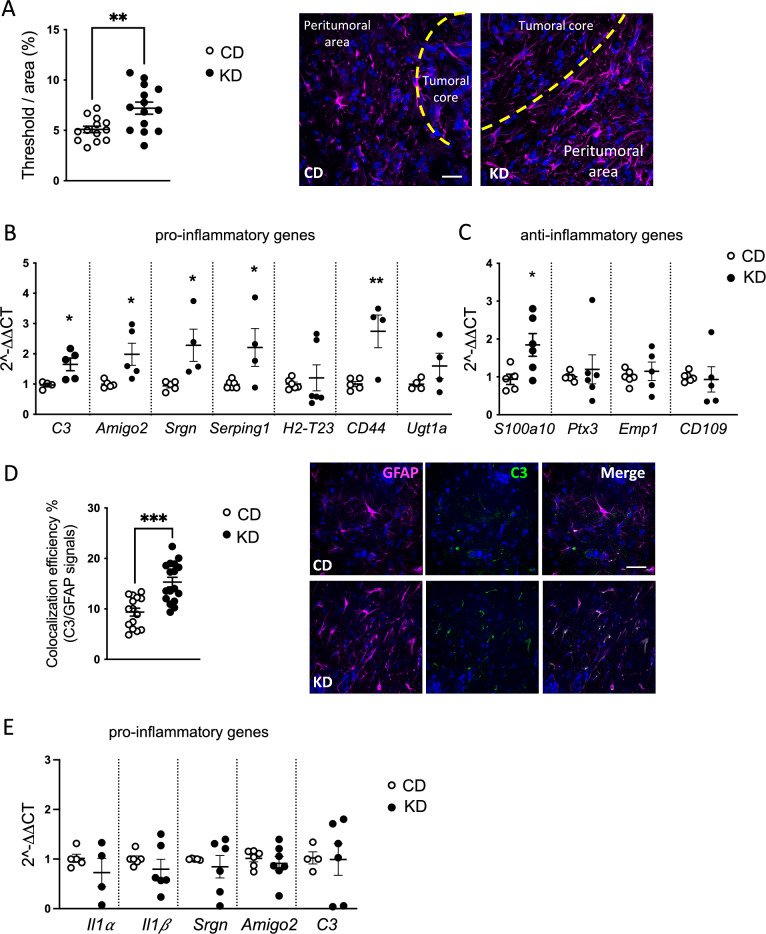
Astrocytes phenotyping upon KD and β-HB administration. **A** Scatter dot plots showing quantification of GFAP^+^ signal expressed as the percentual area occupied by fluorescent staining in CD (n = 13/3 slices/mice) and KD (n = 14/3 slices/mice) mice. Data are presented as the mean ± SEM **p < 0.005, Student’s t-test. Left: Representative z-projection confocal images of GFAP (magenta) in CD and KD tumoral and peritumoral area. Hoechst staining (blu) for nuclei visualization Scale bar: 50 μm. **B** RT-qPCR from ACSA^+^ cells isolated from CD and KD tumoral hemisphere reveals expression of pro-inflammatory genes and (**C**) anti-inflammatory genes. Gene expression is normalized to the housekeeping gene Gapdh. Data are presented as the mean ± SEM n = 4 to 6 mice pulled from two independent experiments. ** p < 0.01; *p < 0.05, Student’s t-test. **D** Scatter dot plots showing the percentual area of GFAP signal colocalizing with C3 staining (Ctrl n = 15/3 slices/mice; KD n = 17/3 slices/mice) Data are presented as the mean ± SEM *** p < 0.001, Student’s t-test. Left: Representative z-projection confocal images of GFAP (magenta) and C3 (green) colocalization in CD and KD peritumor area. Hoechst staining (blu) for nuclei visualization Scale bar: 50 μm. **E** RT-qPCR from Luc^+^-GL261 cells isolated from CD and KD tumoral hemisphere reveals the gene expression level of pro-inflammatory genes. Gene expression is normalized to the housekeeping gene Gapdh. Data are presented as the mean ± SEM n = 4 to 6 mice

Since astrocytes can consume β-HB in cultures and are responsive to this ketone body treatment by increasing the proliferation rate, we investigated the astrocyte reactivity in glioma-bearing mice treated with the KD. By immunofluorescence staining, we describe an increased GFAP staining in the peritumoral area of KD mice respect to CD mice (KD: 7.2 ± 0.6; CD: 5.1 ± 0.32) (Fig. 3A). We further investigated the phenotype of glioma-associated astrocytes, isolating them from the ipsilateral hemisphere of CD and KD mice. Quantitative real-time PCR showed that most of the pro-inflammatory signature genes analyzed (*C3, Amigo2, Srgn, Serping1, and Cd44*), exhibit increased levels of expression in KD mice (Fig. 3B) while only *S100a10* is upregulated among the anti-inflammatory selected genes (Fig. 3C). Moreover, by immunofluorescence staining, we further validate the increased expression of C3 in peritumoral astrocytes of KD mice respect to CD mice (KD: 15.35 ± 0.95, CD: 9.36 ± 0.78) (Fig. 3D).

We confirmed the increased transcription level of pro-inflammatory genes upon β-HB stimulation in primary astrocytes (Fig. S3A). None of the tested anti-inflammatory genes are modulated upon β-HB treatment and, notably, GCM can polarize the astrocyte towards an anti-inflammatory phenotype, as shown by the significantly increased expression level of *S100a10*, *Ptx3*, and *Emp1* transcript (Fig. S3B).

To test the effect of KD on the pro-inflammatory transcriptional profile of glioma cells, we sorted Luc^+^-GL261 cells from mouse brains (Fig. S3C) and found that none of the tested genes were modulated (Fig. 3E), while others, such as *Serping1*, *H2-T23,* and *CD44* were under the limit of detection. These data show that under this dietary regimen, only astrocytes, but not GL261, acquire a pro-inflammatory phenotype.

We also tested the microglia/macrophage phenotype by isolating the CD11b^+^ cells from the ipsilateral hemisphere of CD and KD mice. Quantitative real-time PCR showed that most of the pro-inflammatory signature genes analyzed (Fig. S4A) are significantly upregulated except for *iNOS* whose expression is significantly reduced in parallel with the increased expression of *Arg1* and many other anti-inflammatory genes such as *Chil3*, *Cd206*, *Tgfβ* and *Il10* (Fig. S4B). We further investigated the effect of β-HB on primary microglia cells. Quantitative real-time PCR showed that β-HB is able to increase the transcriptional level of almost all the analyzed genes (Fig. S4C, D). Nevertheless, in the presence of the GCM, the pro-inflammatory genes *IL1β* and *iNOS* are not modulated while anti-inflammatory genes *Chil3* and *Arg1* are significantly up-regulated, thus suggesting that β-HB stimulated microglia acquire a more pronounced anti-inflammatory phenotype in the presence of GCM (Fig. S4D).

Altogether these data demonstrate that astrocytes, directly affected by the KD regimen, exert an anti-tumor function.

### β-HB increases astrocytic glutamate uptake

**Fig. 4:**
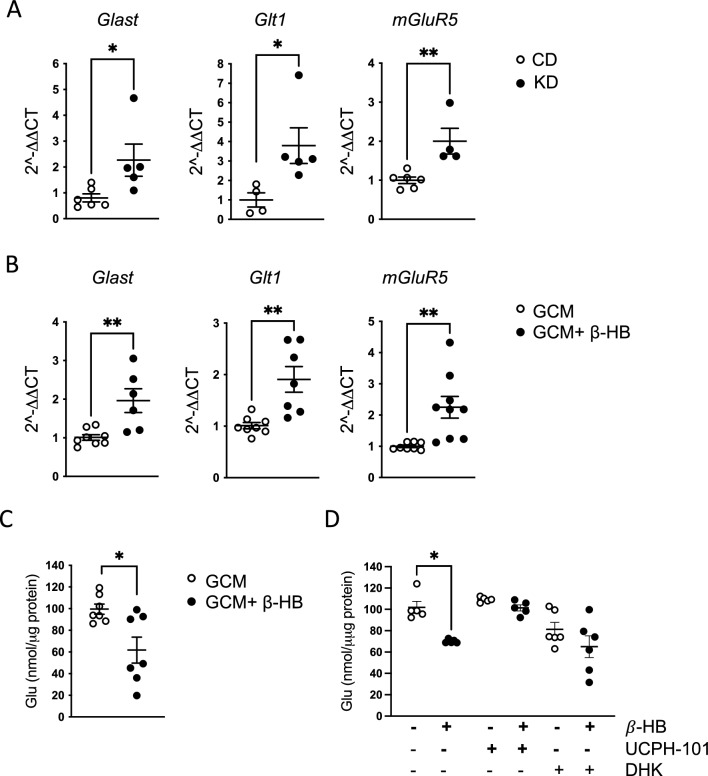
Astrocytic expression of glutamate transporters and glutamate concentration. **A** RT-qPCR from ACSA^+^ cells isolated from CD and KD tumoral hemisphere reveals expression of glutamate transporters Glast, Glt1, and mGluR5 genes. Gene expression is normalized to the housekeeping gene Gapdh. Data are presented as the mean ± SEM n = 4 to 6 mice pulled from two independent experiments. ** p < 0.01; *p < 0.05, Student’s t-test. **B** RT-qPCR from astrocytes cultured in GCM with or without β-HB administration showing the relative expression of glutamate transporters Glast, Glt1, and mGluR5 genes. Gene expression is normalized to the housekeeping gene Gapdh. Data are presented as the mean ± SEM n = 6 to 9 samples pulled from 3 independent experiments. ** p < 0.01 Student’s t-test. **C** Scatter dot plot showing the glutamate quantification in astrocytes cultured in GCM with or without β-HB. Data (nmol/mg of protein) are presented as the mean ± SEM n = 7 samples pulled from 3 independent experiments. * p < 0.05 paired Student’s t-test. **D** Scatter dot plot showing the glutamate quantification in astrocytes cultured in GCM with or without β-HB, stimulated with Glutamate in absence or presence of the UCPH-101 and DHK specific inhibitors of GLAST and GLT-1 transporters. Data (nmol/mg of protein) are presented as the mean ± SEM n = 5–6 samples pulled from 3 independent experiments. * p < 0.05, one-way ANOVA

Since astrocytes can exert an antitumor effect by modulating glutamate levels in the tumor microenvironment, we analyzed the expression of glutamate transporters in astrocytes isolated from CD and KD mice. Quantitative real-time PCR showed that *Glast, Glt1* and *mGluR5* transcripts are upregulated in KD versus CD mice (Fig. 4A) and, in vitro, in GCM-primed astrocytes stimulated with β-HB (Fig. 4B). We further investigated the glutamate transporters functionality by analyzing the residual glutamate concentration in the astrocytic medium, showing a significant reduction in glutamate content upon β-HB treatment (astrocytes + GCM: 100 ± 5 nmol/mg; astrocytes + GCM + β-HB: 62 ± 12 nmol/mg) (Fig. 4C).

Since it has already been shown that GLAST and GLT-1 have a crucial role in astrocytic glutamate uptake [19–21] we investigated the effect of their inhibition on astrocytes upon β-HB exposure.

Astrocytes incubated with an high dose of glutamate also exhibited a reduced concentration of the neurotransmitter when cultivated with β-HB, and this effect is abolished in the presence of the specific GLAST inhibitor, UCPH-101, and GLT-1 inhibitor, DHK, (Fig. 4 D), indicating that these transporters are involved in the β-HB-mediated control of astrocytic glutamate buffering.

### Pro-inflammatory astrocytes and β-HB slow down GL261 proliferation and increase the basal intracellular Ca^2+^ levels

**Fig. 5:**
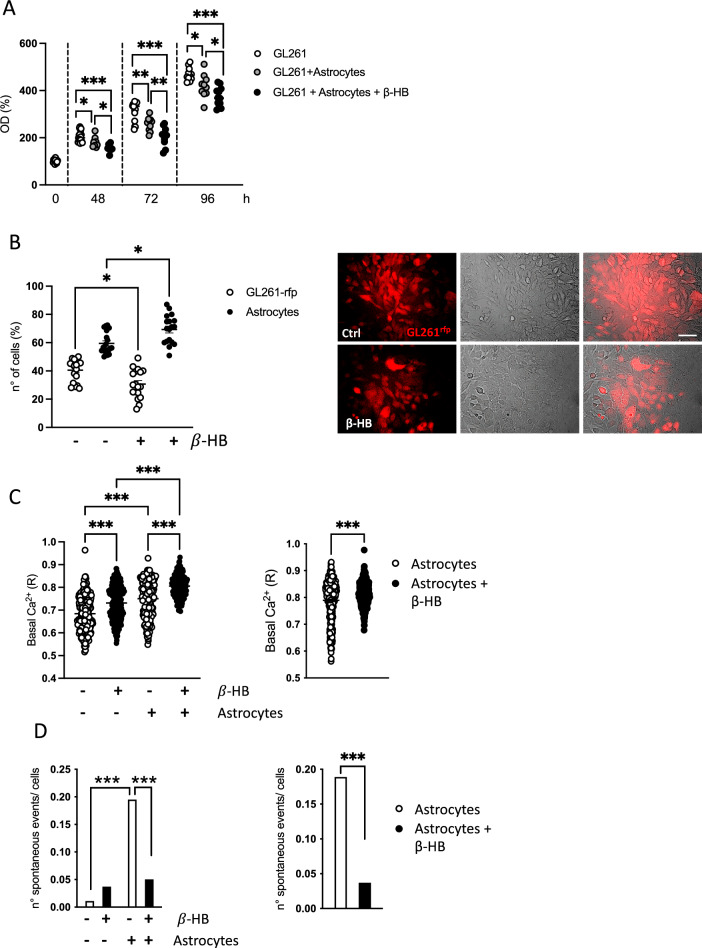
Astrocytes and β-HB effect on GL261 proliferation and spontaneous calcium activity. **A** Scatter dot plot showing the MTT assay on GL261 cells co-cultured with astrocytes and β-HB. Data are presented as the mean ± SEM n = 10 to 18 samples pulled from 3 independent experiments. *** p < 0.001** p < 0.01 *p < 0.05; One-way ANOVA, Tukey’s multiple comparison test. **B** Scatter dot plot showing the percentage of GL261^rfp^ and astrocytes cultured with or without β-HB. Bottom panel: representative immunofluorescence pictures of the direct co-culture between GL26^rfp^ and astrocytes (identified by phase contrast microscope acquisitions, scale bar: 50 μm). Data are presented as the mean ± SEM n = 18–19 analyzed fields of view pulled from n = 3 independent experiments. *** p < 0.001, *p < 0.05; Two-way ANOVA, Fisher’s LSD test. **C** Left panel: Scatter dot plot showing the effect of β-HB and astrocytes on the basal Ca^2+^ level of GL261^rfp^. Data are presented as the mean ± SEM n = 178–260 cells analyzed from 3 independent experiments. *** p < 0.001; Two-way ANOVA, Fisher’s LSD test. Right panel) Scatter dot plot showing the basal Ca^2+^ level of astrocytes co-cultured with GL261^rfp^ and stimulated with vehicle or β-HB. **D** Left panel: Bar graph showing the number of spontaneous events recorded in GL261^rfp^ co-cultured or not with astrocytes, with or without β-HB stimulation. (*** p < 0.001) Right panel: Bar graph showing the number of spontaneous events recorded in astrocytes co-cultured with GL261.^rfp^ and stimulated with vehicle or β-HB. (*** p < 0.001)

To test whether astrocytes counteract GL261 growth upon BHB exposure, we co-cultured astrocytes with glioma on a transwell system and measured GL261 proliferation. The presence of astrocytes significantly reduced GL261 cell proliferation and β-HB further depressed it (Fig. 5A). We also tested the effect of microglia on the GL261 proliferation: microglia conditioned media (MCM) reduced the GL261 proliferation rate, without further effect mediated by β-HB (Fig. S5).

The presence of β-HB in the co-culture of GL261 cells and astrocytes changes their proliferation: after 48 in the presence of β-HB, the mixed GL261^rfp^/astrocytes co-cultures showed a reduced percentage of GL261^rfp^ and an increased percentage of astrocytes (Fig. 5B). A further evidence of the effect of β-HB-treated astrocytes on glioma cells came from the analysis of intracellular free Ca^2+^ concentration ([Ca^2+^]_i_). While β-HB induced a small but significant increase in basal [Ca^2+^]_i_ in GL261^rfp^, when in co-culture with astrocytes, we measured a larger increase in GL261^rfp^ [Ca^2+^]_i_, and this effect was further enhanced in the presence of β-HB (Fig. 5C left). β-HB increased basal [Ca^2+^]_i_ also in astrocyte monocultures (Fig. 5C right). Furthermore, astrocytes enhanced the frequency of spontaneous Ca^2+^ transients in GL261^rfp^, an effect strongly inhibited by β-HB treatment (Fig. 5D left panel). The same β-HB effect was observed in astrocyte monoculture (Fig. 5D right panel), suggesting that spontaneous Ca^2+^ transients in astrocytes might regulate the same activity in GL261 cells. Taken together these data indicate that β-HB-treated astrocytes can modulate the Ca^2+^ homeostasis of GL261 cells.

## Discussion

In the last decade, the ketogenic diet represented a novel adjuvant approach to standard glioma treatment by targeting the altered metabolism characteristic of cancer cells [22–28].

While preclinical studies and some clinical case reports have shown promising results for the use of KD in glioma treatment, the effects on cancer and brain cells are still largely unknown. In addition to the complexities surrounding the metabolic interactions within glioma cells, the pieces of information regarding the role of glial cells in the context of the KD and the potential therapeutic effects are far to be understood. Glial cells play critical roles in maintaining brain homeostasis and in responding to pathological conditions such as cancer [29], influencing tumor progression and responses to therapeutic treatment through various mechanisms, including the immune-metabolic crosstalk [18, 28] and the remodeling of tumor microenvironment [31, 32].

Here, we investigated how a restricted KD in adult mice is able to reduce tumor growth and increase mice survival. Our data are in line with several research papers showing the efficacy of the KD in counteracting glioma growth [5, 33, 34]. We further explored the role exerted by β-HB, the major ketone body produced during ketosis, in glioma-bearing mice. The reduced tumor volume observed in these mice not only reveals that β-HB is a potent mediator of KD but also poses it as a putative alternative to this restrictive diet, often poorly tolerated by patients [25, 35, 36].

Besides the increased level of β-HB, we observed reduced blood glucose levels in KD mice respect to CD mice. In addition, we found that there is a positive correlation between the glycemic levels and tumor volumes. This, together with the alteration of glucose level in the blood of glioma-bearing mice, suggest that high glucose could be predictive of a worse prognosis in tumor-grafted mice. This result is in line with other studies showing a positive correlation between hyperglycemia and the Ki67 index in GBM specimens [37] and poorer overall survival in GBM patients [38]. Our data demonstrated that the development of a hyperglycemic phenotype is linked to tumor progression independently of the type of diet. Future studies will be necessary to better investigate the mechanisms responsible of this effect.

Since glioma cells are not able to utilize ketone bodies for energy production [5], and since the peritumoral area, is the main target of adjuvant therapies, it is important to deepen our knowledge of the cellular and molecular mechanisms of tumor-glia interplay.

Here, we investigated the potential role played by glial cells in counteracting glioma progression upon KD or β-HB treatment. We first showed that GL261 cell proliferation is not affected by β-HB treatment per se and that these cells are not consuming ketone bodies in vitro, one evidence supported by the increased concentration of β-HB in the tumor tissue of KD mice [5] and by the absence of intracellular β-HB in GL261 cells. Nevertheless, we cannot exclude that this result is due to a rapid uptake and release kinetics of β-HB by tumor cells.

In contrast, both astrocytes and microglia consume β-HB, which promotes their metabolic reprogramming [10, 39–41] and differently modulates the level of monocarboxylate transporters and β-HB converting enzymes.

In addition to the effect on cellular metabolism, KD metabolic products can influence many different cellular functions such as gene expression, oxidative stress, and inflammation in physiological and pathological conditions [11].

We investigated the effect of KD and β-HB on the tumor-associated glial cells, describing the phenotype acquired by astrocytes and microglia. It has been reported that astrocytes surrounding gliomas upregulate GFAP expression (42, 43) and that tumor-associated astrocytes and microglia acquire an anti-inflammatory/pro-tumoral state [15, 42]. We characterized the transcriptional changes of the signature genes representative of pro-inflammatory and anti-inflammatory glial cells isolated from the tumoral hemisphere of KD respect to CD mice. Astrocytes isolated from the tumoral hemisphere of glioma-bearing mice revealed that KD up-regulated almost all the tested pro-inflammatory genes, together with C3, particularly enriched in inflammatory astrocytes [44–46].

We further showed the in vitro effect of β-HB on astrocytes, switching the anti-inflammatory phenotype induced by the presence of GCM towards a pro-inflammatory phenotype. This effect, mediated by the KD and its metabolic product, further underlines that tumor-associated anti-inflammatory astrocytes aid the evolution of an immunosuppressive environment in glioblastoma [42] and that tumor-associated pro-inflammatory astrocytes are effective in counteracting glioma growth.

A crucial role of inflammatory mediators in glioma biology has been described for TNFα [47, 48]. In addition to TNFα, also microglial derived IL1α has been proposed as a potent inducer of pro-inflammatory astrocytes [49]. In line with this evidence, we reported that the CD11b^+^ cells isolated from KD mice exhibit increased levels of pro-inflammatory mediators potentially involved in the induction of pro-inflammatory astrocytes.

Although it has been shown that β-HB and the shift of microglial glucose metabolism from glycolysis to oxidative phosphorylation is associated with an anti-inflammatory phenotype [14, 50, 51], in glioma-bearing mice, the microglia/macrophages cell profile reveals that KD is inducing a mixed activation state that cannot be ascribed to the old-fashion pro-inflammatory or anti-inflammatory classification.

Glial cells play important roles during brain tumor progression influencing cancer invasion [52–55], aggressiveness, and resistance to therapies [56]. In particular, peritumoral astrocytes are less effective in buffering the glutamate extracellular concentration thus orchestrating the peritumoral neuronal hyperexcitability and excitotoxic death [43].

Considering the beneficial effect mediated by the KD, we further investigated the functional role of astrocytes in buffering extracellular glutamate in the tumor microenvironment. We report an increased expression of the metabotropic glutamate receptor mGluR5 and of the glutamate transporters Glt-1 and Glast, which tightly regulate the concentration of the excitatory neurotransmitter in the extracellular space [57, 58]. Moreover, we observed that astrocytes, in the presence of β-HB, are functionally active in reducing the extracellular glutamate and that the blockade of GLAST and GLT-1 reverted the β-HB effect. These results indicate these transporters as possible mediators of the KD efficacy in decreasing neuronal hyperexcitability [59, 60], thus suggesting a commitment of peritumoral astrocytes to a neuroprotective effect on brain parenchyma [61]. However, further studies will be necessary to better understand the role played by astrocytes in the β-HB-dependent effect on glioma.

To investigate the potential involvement of pro-inflammatory astrocytes in reducing tumor growth, taking advantage of a co-culture system, we observed that astrocytes mediate a reduction in glioma proliferation and that the presence of β-HB further enhances this effect. This supports the notion that astrocytes surrounding glioma can restrain tumor proliferation [62] and that increased tumor cell apoptosis could be induced also by increasing astrocytes glutamate uptake capability [63, 64].

Further studies using additional experimental approaches (including a more detailed analysis of β-HB metabolism and pharmacological inhibition of the acquisition of a proinflammatory phenotype in astrocytes) will be necessary to better understand the role of β-HB in mediating the mechanisms of glial cell activation that interfere with tumor proliferation.

In direct co-culture experiments, we observed an astrocyte-driven reduction of glioma proliferation along with an increase in GL261 basal [Ca^2+^]_I,_ and we reported that these effects were further increased by β-HB. β-HB-treated astrocytes dysregulate Ca^2+^ homeostasis of glioma cells, likely leading to glioma cell dysfunction, potentially linked to the observed inhibition of tumor growth [65].

In conclusion, we demonstrated that KD and β-HB induce a pro-inflammatory phenotype of glioma microenvironment reducing tumor cell proliferation, increasing intracellular basal Ca^2+^ level, and promoting neuroprotection by reducing extracellular glutamate level mediated by astrocytic glutamate uptake.

## Materials and methods

### Animal husbandry

C57BL/6N mice were housed (two to four animals for each cage) under a 12-h light cycle in standard cages and fed ad libitum with a standard diet (4RF21 Mucedola s.r.l.). They were randomly allocated to the different experimental groups. All the experiments in this study were conducted seeing the ARRIVE guidelines [66], and were approved by the Italian Ministry of Health (authorization No.775/2020-PR) following the EC Council Directive 2010/63/EU and the Italian d.lgs.26/2014. All the efforts were done to reduce animal suffering, and to reduce the number of animals, estimating the necessary sample size before starting the experiments.

### Tumor cell implantation, diet composition and administration

Glioma syngeneic mouse models (GM) are obtained by injecting glioma cells (GL261) 1 × 10^5^ cells at 2 mm lateral, 1 mm anterior to the bregma, and 3 mm depth in the right striatum. Two days after tumor implantation, the mice are randomly assigned to two/three groups: standard diet (SD), control diet (CD), and the ketogenic diet (KD) group, and start to be fed with the different diets (Mucedola s.r.l. for CD and KD, Altromin as SD) for three weeks after tumor implantation. The diets composition are reported in Table 1. The KD diet has a caloric density of 6.73 kcal/gr and each animal was fed with 1–3 g/day. The body weight is constantly monitored throughout the experimental setup to avoid a weight loss of 10–15% higher than the starting point of treatment. Control mice received an isocaloric amount of food compared to the ketogenic diet. For survival analysis glioma injected mice were daily monitored and the endpoints were determined by lack of physical activity or 20% weight loss. The mean survival time was calculated using the Kaplan–Meier method and statistical analysis was performed using a log-rank test.

### In-vivo β-HB administration

Two days after tumor implantation, the mice are intraperitoneally injected with 50 µl of saline or β-HB (120mg/kg; Fisher Scientific 150-83-4), every other day. Three weeks after tumor implantation mice are sacrificed and brains are analyzed for tumor volume evaluation. During the entire experiment mice are fed ad libitum with a standard diet.

### Tumor volume evaluation

Sequential 20 μm coronal brain slices (one each 100 μm of the entire tumor length) were collected and stained by standard H&E protocol. Tumor volume was calculated via the formula (volume = *t* ×$\sum$ *A*), where *A* is the tumor area/slice and *t* is the thickness by ImageTool3.0 software.

### Isolation of mouse astrocytes, CD11b^+^ cells, and RNA extraction

Glioma-bearing mice were anesthetized and perfused with PBS. Brains were removed, the tumor portion is isolated from the ipsilateral hemisphere and a single-cell suspension was obtained in Hank's balanced salt solution (HBSS) by mechanical dissociation using a glass wide-tipped pipette. The cell suspension was applied to a 30 μm cell strainer (Miltenyi Biotec). Cells were processed instantly for MACS MicroBead separation. ACSA2^+^ cells or CD11b^+^ cells were magnetically labeled with the respective MicroBeads (Miltenyi Biotec 130097678; 130049601). The cell suspension was loaded onto a MACS Column placed in the magnetic field of a MACS Separator and the negative fraction was collected. After removing the magnetic field, ACSA2^+^ and CD11b^+^ cells were eluted as a positive fraction. Total RNA was isolated with Tri-Reagent (Merck), and processed for real-time PCR. The quality and yield of RNAs were verified using the NANODROP One system (Thermo Scientific).

### Immunofluorescence staining on brain tissue and image acquisition

By using 3% isofluorane mice were anesthetized and perfused transcardially with cold PBS and 4% PFA in 0.1 M PB. Brains were quickly removed, stayed overnight in PFA 4%, washed with PB, and cryoprotected in PB 30% sucrose solution. We then collected 20 μm- thick coronal sections by using cryostat microtome (Leica Microsystems) at 20 °C. Briefly, slices were immersed for 30 min in a boiling citrate buffer solution for antigen retrieval, then incubated with blocking solution (0.5% Triton X-100, 5% BSA) for 1 h at RT. Sections were incubated with primary antibodies (Ki67 M3063 SP6 clone Spring Bioscience 1:50; 1:100; GFAP MAB30060 Millipore 1:200; C3 A0063 Dako 1:50) in diluted blocking solution overnight at 4 °C and 1 h at RT with fluorophore-conjugated secondary antibodies (Alexa Fluor 594 goat anti-rat) and Hoechst for nuclei visualization. The sections were mounted with a fluorescence mounting medium (DAKO) or with a ProLong Glass Antifade Mountant (Thermo Fisher).

Images were collected with spinning disk confocal microscopy on an Olympus IX73 microscope equipped with X-Light V3 spinning disk (CrestOptics), LDI laser source and a Prime BSI Scientific CMOS (sCMOS), 6.5 µm pixels (Photometrics) with a 60x/1.4 PlanApo l oil objective and 10 × objectives. The Z step size was 1 µm and 0.5 µm respectively for 10X and 60X objectives. All the images were acquired by using Metamorph software version 7.10.2. (Molecular Devices, Wokingham, UK) and then analyzed with ImageJ or Metamorph software.

### Cell lines and primary glial culture

Glioma cells GL261, are maintained in DEMEM 20% FBS, CT2-a, U87 and U373 cell lines are cultured in DMEM 10% FBS. Cortical primary glial cells were prepared from 0- to 2-d-old mice: cerebral cortices were chopped and digested in 30 U/ml papain for 40 min at 37 °C followed by gentle trituration. The dissociated cells were washed, suspended in DMEM with 10% FBS (Invitrogen) and 2 mM L-glutamine, and plated at a density of 9–10 × 10^5^ in 175 cm^2^ cell culture flasks. At confluence (10–14 DIV), glial cells were shaken for 2 h at 37 °C to detach and collect microglia and astrocyte cells. After seeding, cells were cultured in DMEM low glucose (D6064, Sigma-Aldrich) treated for the indicated time point with β-HB (Sigma Aldrich, H6501, 10 mM) in the presence of a glioma-conditioned medium (GCM), or co-cultured with GL261 cells. In co-culture experiments, GL261 cells (1.5 × 10^4^ cells) were seeded in the lower compartment of a 24-well transwell® system (Sigma Aldrich, 3413, 0.4 µm pore size) containing astrocytes (3 × 10^4^ cells). For direct co-culture, GL261 RFP cells (1.5 × 10^4^) were mixed with astrocytes (3 × 10^4^ cells), and seeded on tissue culture dishes (Corning Falcon, 353001, 35 mm). In all these experiments β-HB is administered at a final concentration of 10 mM.

### Human primary cultures

The human primary cells are obtained as previously reported [67]. Briefly, the surgical resection is reduced to small pieces under a lamina flow hood, and the addition of DMEM facilitates the generation of a cell suspension that is filtered through a 100 μm cell strainer. The cell suspension is centrifuged at 800 rcf for 5 min; the supernatant is removed and, if the pellet is partially made of red blood cells, hemolysis is performed with Red Blood Cells Lysis Buffer (0.154 M Ammonium Chloride, 10 mM Potassium Carbonate, 0.1 M EDTA, double-distillated water, pH 7.4). The pellet is resuspended in the buffer at 4 °C; after 3 min the cell suspension is centrifuged at 800 rcf for 5 min. The supernatant is removed, the pellet is washed with PBS^−/−^ and centrifuged again at 800 rcf for 5 min. The pellet is resuspended in DMEM and seeded in a flask accordingly with the dimensions of the dissected piece. After 24 h in DMEM without serum, the medium is changed to 10% FBS DMEM.

### Plasmid generation and GL261 stable transfection

The epB-BSD-PGK-FLuc plasmid was obtained by replacing the tagRFP coding sequence of the enhanced piggyBac transposable vector ePB-BSD-PGK-tagRFP [68] with the Firefly luciferase (FLuc) coding sequence (1678 bp), which had been PCR-amplified from the psiCHECK-2 vector (Promega) using the following primers: LuciferaseF FW 5′-CATGGATCCACCATGGCCGATGCTAAGAA-3′ and LuciferaseF RV 5′-CATGCGGCCGCTATTACACGGCGATCTTGCC-3′ and cloned into the BamHI and NotI restriction sites. GL261 cells were stably co-transfected with 1.5 μg of epB-BSD-PGK-FLuc plasmid and 150 ng of the piggyBac transposase using Lipofectamine 2000 (Life Technologies). Stably transfected cells were obtained by selection for 10 days in 2 μg/ml blasticidin.

### Conditioned medium preparation

GCM is collected from GL261 (1 × 10^6^ cells in 1 ml of DMEM -FBS). Microglia cells were also treated with β-HB (10 mM) in the presence of a glioma-conditioned medium (GCM) and microglia-conditioned medium (MCM) was also collected (2 × 10^4 ^cells in 1 ml of DMEM-FBS).

### Glucose and β-HB measurement

The evaluation is performed through a detection device (Wellion-Galileo) that uses strips for the detection of glucose and β-HB in blood and from GL261, microglia, and astrocyte cell medium treated with β-HB.

### MTT assay

MTT assays have been performed on: GL261 murine glioma, CT2a, U87, U373 and GBM206 cells treated with different concentrations of β-HB, GL261 cells co-cultured with astrocytes or in the presence of microglia-conditioned media (MCM), astrocytes and microglia alone or in the presence of GCM. After being treated with β-HB (10 mM) for 24, 48 h, 72 h, and 96 h, as specified for each experiment, MTT (dissolved in PBS with a final density of 0.5 mg/ml) was added to the medium culture. After 90 min incubation, the MTT solution was extracted, mixed with DMSO, and shaken for 20 min. Finally, the absorption of the samples was read by regulating the 570-nm filter as the main wavelength and the 630-nm filter as the referenced wavelength. Blank was subtracted from all samples to obtain pure cellular absorption.

### Measurement of intracellular β-HB

Astrocytes are seeded in T25 flask (2 × 10^6^ cells per flask) in 10% FBS DMEM. The next day the medium is removed and Low Glucose DMEM without FBS is added to the flasks; after 1 h of starvation, β-HB 10 mM is administered to all the flasks for 30 min. After β-HB administration cells are harvested and centrifuged at 800 rpm for 7 min; the supernatant is removed and the pellet is lysed at T0, and after 10 and 30 min in the lysis buffer from β-HB assay kit (ab83390, Abcam). When all the cells are lysed, the colorimetric assay is performed according to the manufacturer’s instructions.

### Real-time PCR

Samples were lysed in TRYzol reagent for the isolation of total RNA. The quality and yield of RNAs were verified using NANODROP One (Thermo Scientific). For RT-PCR one microgram of total RNA was reverse transcribed by using ThermoScript RT-PCR System. RT-PCR was carried out using Sybr Green (Bio-Rad) according to the manufacturer’s instructions. The PCR protocol consisted of 40 cycles of denaturation at 95 °C for 30 s and annealing/extension at 60 °C for 30 s. For quantification, the comparative Threshold Cycle (Ct) method was used. The Ct values from each gene were standardized to the Ct value of GAPDH in the same RNA samples. Relative quantification was performed using the 2^−∆∆Ct^ method and expressed as fold change in arbitrary values.

### Fluorescence-activated cell sorting of Luc.^+^-GL261

Luc^+^-GL261 glioma-bearing mice were anesthetized and brains were removed, the tumor portion is isolated from the ipsilateral hemisphere and a single-cell suspension was obtained in Hank's balanced salt solution (HBSS) by mechanical dissociation using a glass wide-tipped pipette. The cell suspension was applied to a 30 μm cell strainer (Miltenyi Biotec) and myelin was removed with 30% percoll (P4937 Sigma-Aldrich) gradient. Before the sorting cells are incubated with luciferin (150 µg/ml) for 5 min and sorted based on luciferase expression level using a BDFACSAriaIII (BD Biosciences) equipped with a 488 nm laser, and FACSDiva software (BD Biosciences version 6.1.3). Data were analyzed using FlowJo software (Tree Star, version 10.10.0). Briefly, cells were first gated based on morphology using forward versus side scatter parameters (FSC-A versus SSC-A) were then doublets excluded considering morphology parameter area versus width (A versus W). Cells were then detected in the green fluorescence channel for luciferase expression (530/50 nm filter) and collected as Luc + . To reduce stress, cells were isolated in gentle sorting conditions using a ceramic nozzle of size 100 µm, a low sheath pressure of 19.84 pound-force per square inch (psi) that maintains the sample pressure at 18.96 psi and an acquisition rate of 1000 events/s. Cells were collected in 1.5 mL Eppendorf tubes and later on RNA was extracted. Upon cell sorting, an aliquot of each tube with isolated cells was evaluated for purity at the same instrument resulting in a good enrichment.

### Glutamate assay

Primary murine astrocytes were plated in 24-well plates (2 × 10^5^ cells/well) and treated with GCM or with GCM + β-HB (10 mM). After 72 h, the cell medium was collected and centrifuged at 10 000 g × 15’ to remove insoluble material. Glutamate concentration was measured by a colorimetric assay, according to the manufacturer’s instructions (MAK004 Sigma-Aldrich). The cells were lysed in RIPA buffer and the protein content of each well was quantified by BCA assay (Pierce) and used for normalization.

For the GLAST pharmacological inhibition, astrocytes were treated as indicated before with GCM or with GCM + β-HB (10 mM). After 72 h, the cell medium was removed and cells were washed in Locke's modified buffer (in mM: 154NaCl, 5.6 KCl, 3.6 NaHCO_3_, 5 HEPES, 2.3 CaCl_2_, 5.6 glucose, 10 glycine pH 7.4) and incubated in the same buffer with glutamate (100 µM, 30 min) with or without UCPH-101 (4390 Tocris Bioscience, 25 µM for the specific GLAST blockade) and DHK (0111 Tocris Bioscience, 25 µM for the specific GLT-1 blockade). The stimulation medium was collected and centrifuged at 10,000 g × 15’ to remove insoluble material. Glutamate concentration was measured by a colorimetric assay, according to the manufacturer’s instructions (MAK004 Sigma-Aldrich). Protein extract from RIPA buffer lysis is quantified by BCA assay (Pierce) and used for normalization.

### Calcium imaging

Changes in free intracellular Ca^2+^ concentration [Ca^2+^]_i_ was quantified by time-resolved digital fluorescence microscopy using the Ca^2+^ indicator Fura-2 (excitation 340 nm and 380 nm, emission 510 nm). The changes of [Ca^2+^]_i_ were expressed as R = F_340_/F_380_. GL261 and astrocyte cultures were incubated with the cell-permeant Fura-2 acetoxymethyl ester (2 μM; Molecular probes, Life technologies) for 1 h at 37 °C in culture medium. Cells were then washed and placed in normal external solution (NES) for fluorescence microscopy experiments. [Ca^2+^]_i_ variations with ΔR > 0.01 were considered as spontaneous activity. Cells were continuously perfused during the experiment. [Ca^2+^]_i_ variations were measured separately from each individual cell, using the MetaFluor 7.0 software (Molecular Devices, USA). NES contained (in mM): 140 NaCl, 2.8 KCl, 2 CaCl2, 2 MgCl2, 10 glucose, and 10 HEPES; pH value adjusted with NaOH at 7.3.

### Statistical analysis

The repeat (n) for each experiment and details of statistical analyses are described in the figure legends or main text. Data are reported as mean ± SEM. Statistical analysis, normality tests, and non-parametric tests were performed, when appropriate, with GraphPad Prism 9 software. The exact p-values are indicated in the text where available and the multiplicity-adjusted p-values are indicated in the corresponding figures (*p < 0.05, **p < 0.01, ***p < 0.001). Paired T-test is used to compare tumor volume and glutamate measurement, and an unpaired T-test is used for immunofluorescence analysis. For real-time PCR an unpaired T-test or a Two-way ANOVA, Fisher’s LSD test was run to determine significant differences for the considered genes. For MTT assays a Mann–Whitney U test was run to determine significant differences for the considered experimental conditions.

Basal Ca^2+^ values were expressed as basal R values (means ± S.D.) and analyzed using one-way ANOVA test. When necessary, the non-parametric Dunn’s one-way ANOVA on ranks was used. In case of significance, all pairwise multiple comparison procedure was used (Holm-Sidak, or Dunn’s method for non-parametric tests). Responsive cells data were expressed as number of spontaneous event/cell and analyzed using χ^2^ test. The minimum power of statistical tests was set at 0.8. The significance for all tests was set at p < 0.05.

## Supplementary Information

Below is the link to the electronic supplementary material.

Supplementary file1 (DOCX 4913 KB)

## Data Availability

The data analyzed during this study are included in this published article and the supplemental data files. Additional supporting data are available from the corresponding authors upon reasonable request.
