# Supplementary material for: Ketogenic diet induces an inflammatory reactive astrocytes phenotype reducing glioma growth
Source: Cell Mol Life Sci. 2025 Feb 8;82(1):73. doi: 10.1007/s00018-025-05600-4 (PMC11807044; doi:10.1007/s00018-025-05600-4)
Supplement: Supplementary file 1 — Supplementary file1 (DOCX 4913 KB) [file 18_2025_5600_MOESM1_ESM.docx]

**Supplementary material**

**Ketogenic diet induces an inflammatory reactive astrocytes phenotype reducing glioma growth**

**Journal: Cellular and molecular life science**

Maria Rosito^1,2*^, Javeria Maqbool^1^, Alice Reccagni^1^, Micol Mangano^1^, Tiziano D’Andrea^3^, Arianna Rinaldi^1^, Giovanna Peruzzi^2^, Beatrice Silvestri^2,4^, Alessandro Rosa^2,4^, Flavia Trettel^1^, Giuseppina D’Alessandro^1,3^, Myriam Catalano^1^, Sergio Fucile^1,3^, Cristina Limatola^3,5 *^ .

^1^Department of Physiology and Pharmacology, Sapienza University, Rome, Italy

^2^ Center for Life Nanoscience & Neuroscience Istituto Italiano di Tecnologia@Sapienza, Rome, Italy;

^3^IRCCS Neuromed, Pozzilli, IS, Italy;

^4^Department of Biology and Biotechnologies "Charles Darwin", Sapienza University of Rome, Rome, Italy.

^5^Department of Physiology and Pharmacology, Sapienza University, Laboratory affiliated to Institute Pasteur Italia – Rome, Italy.

* Corresponding authors:

Cristina Limatola, Department of Physiology and Pharmacology, Sapienza University, P.le Aldo Moro 5, 00185, Rome, Italy; [cristina.limatola@uniroma1.it](mailto:cristina.limatola@uniroma1.it)

Maria Rosito, Department of Physiology and Pharmacology, Sapienza University, P.le Aldo Moro 5, 00185, Rome, Italy; [maria.rosito@gmail.com](mailto:maria.rosito@gmail.com)

**Fig.S1: Effect of the ad libitum feeding on tumor growth and body weight**

**A)** Bar plot showing tumor size in SD, CD and KD mice, *n* = 5-6. Data are presented as the mean ± SEM *p <0.05, One-way ANOVA - Dunnett’s multiple comparison test. Lower pictures: Representative images of brain coronal slices, scale bar = 1 mm. **B)** Body weight measurements of SD, CD and KD GL261 bearing mice. *n* = 5-6 Data are presented as the mean ± SEM. SD vs KD: **^§^** p < 0.05; CD vs KD: ** p < 0.005; *** p < 0.001. One-way ANOVA – Tukey’s multiple comparison test, evaluated on days 6, 14, and 20.

**Fig. S2: Transcriptional expression of monocarboxylate transporters and ketolytic enzymes**

**A)** Bar plot showing the MTT assay on CT2a cells stimulated with different β-HB concentrations Data are presented as the mean ± SEM *** p < 0.001. One-way ANOVA - Dunnett’s multiple comparison test versus time 0. **B)** RT-qPCR from GL261 mRNA. Gene expression of Mct2 and Mct4 is normalized to the housekeeping gene Gapdh, n = 6 to 7 samples pulled from 3 independent experiments. Data are presented as the mean ± SEM. **C)** RT-qPCR from GL261 mRNA. Gene expression of Bdh1, Bdh2, and Oxct1 is normalized to the housekeeping gene Gapdh, n = 6 to 7 samples pulled from 3 independent experiments. Data are presented as the mean ± SEM. **D)** RT-qPCR from astrocytes mRNA. Gene expression of Mct2 and Mct4 and **E)** Bdh1, Bdh2, and Oxct1 are normalized to the housekeeping gene Gapdh, n = 6 to 7 samples pulled from 3 independent experiments. Data are presented as the mean ± SEM. **F)** RT-qPCR from microglia mRNA. Gene expression of Mct2 and Mct4 and **G)** Bdh1, Bdh2, and Oxct1 are normalized to the housekeeping gene Gapdh, n = 4 to 7 samples pulled from 3 independent experiments. Data are presented as the mean ± SEM * p < 0.05 ** p < 0.005 *** p < 0.001 Two-way ANOVA. Fisher’s LSD test.

**Fig. S3: Astrocytes phenotyping upon β-HB administration**

**A)** RT-qPCR from astrocytes with or without β-HB and GCM administration showing the relative expression of pro-inflammatory and **(B)** anti-inflammatory genes. Gene expression is normalized to the housekeeping gene Gapdh. Data are presented as the mean ± SEM n = 7 to 16 mice pulled from 4 independent experiments. *** p <0.001** p <0.01; Two-way ANOVA, Fisher’s LSD test.

**C)** Gate strategy for isolation of glioma cells based on luciferase expression level. Luciferase positive cells were sorted according to the represented gating strategy for a representative sample. Dot plot show an initial glioma cell population (4%) isolated for luciferase expression levels (FITC signal, 68%). Green dot plot on the left show the final purity of the sorted cells.

**Fig. S4: Microglia/macrophages phenotyping upon KD and β-HB administration**

**A)** RT-qPCR from Cd11b^+^ cells isolated from CD and KD tumoral hemisphere reveals transcriptional expression levels of pro-inflammatory and **B)** anti-inflammatory genes. Gene expression is normalized to the housekeeping gene Gapdh. Data are presented as the mean ± SEM n = 4 to 8 mice pulled from two independent experiments. ** p <0.01; *p <0.05, Student’s t-test. **C)** RT-qPCR from primary microglia with or without β-HB and GCM administration showing the relative expression of pro-inflammatory and **D)** anti-inflammatory genes. Gene expression is normalized to the housekeeping gene Gapdh. Data are presented as the mean ± SEM n = 4 to 9 samples pulled from 3 independent experiments. *** p <0.001** p <0.01 *p <0.05; Two-way ANOVA, Fisher’s LSD test.

**Fig. S5 Effect of MCM and β-HB on GL261 proliferation**

**A)** Scatter dot plot showing the MTT assay on GL261 cells in the presence of MCM and β-HB. Data are presented as the mean ± SEM n = 16 to 18 samples pulled from 3 independent experiments. ** p <0.01 *p <0.05; One-way ANOVA, Tukey’s multiple comparison test.

Rosito et al., Fig. S1


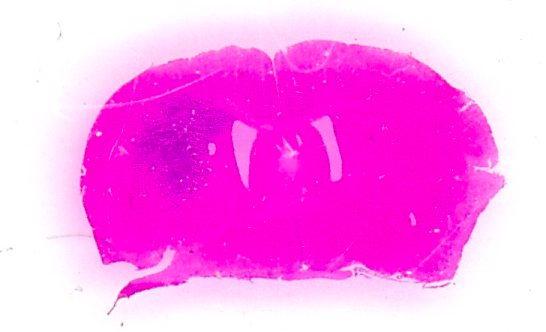

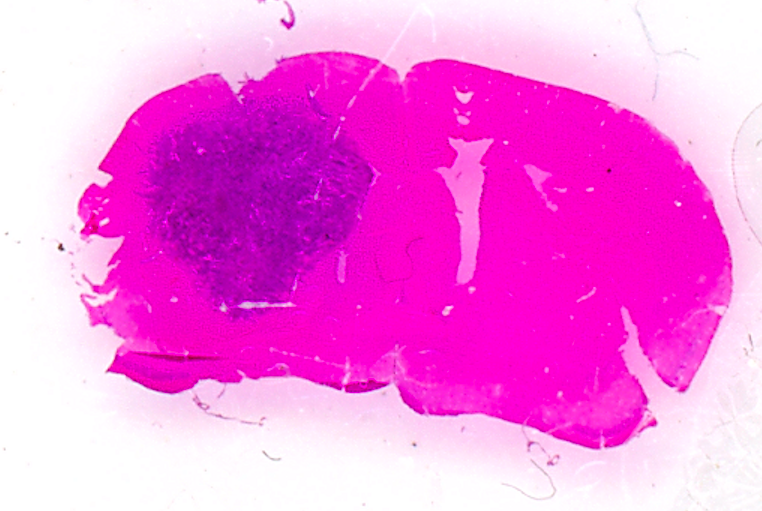

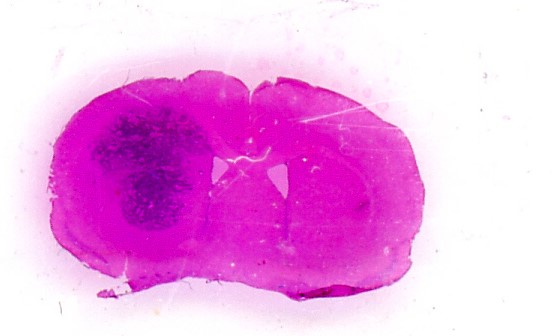


Weeks of diet

BW (gr)

A

B

*Mct2 Mct4*

**- + - + - + - + 𝛽-HB**

**- - + + - - + +** GCM

A

B

*Bdh1 Bdh2 Oxct1*

Ctrl

β-HB

*Mct2 Mct4*

**- + - + - + - + - + - + 𝛽-HB**

**- - + + - - + + - + - +** GCM

*Bdh1 Bdh2 Oxct1*

C

D

E

G

F

*Mct2 Mct4*

*Bdh1 Bdh2 Oxct1*

**- + - + - + - + 𝛽-HB**

**- - + + - - + +** GCM

0 48 72 96 h

CT2a

**- + - + - + - + - + - + 𝛽-HB**

**- - + + - - + + - + - +** GCM

GL261

Astrocytes

Microglia

GL261

Astrocytes

Microglia

Rosito et al., Fig. S2

**SSC**

**FITC**

**SSC**

**FSC**

**SSC**

**FITC**


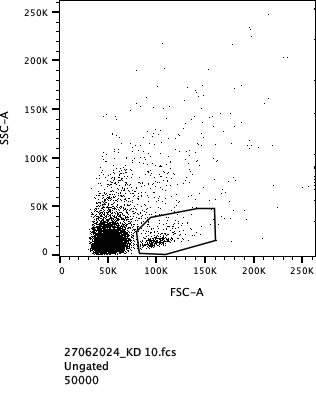

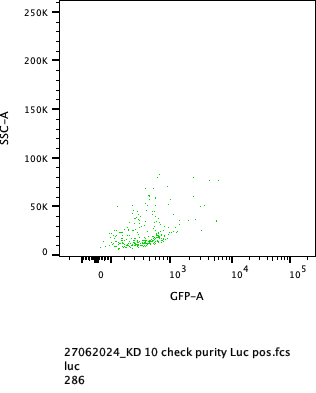


Luc+

4%


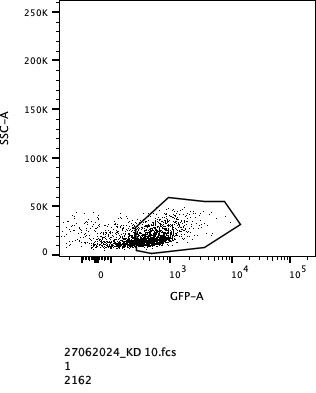


luc+ cells

68%

*S100a10 Ptx3 Emp1*

**- + - + - + - + - + - + 𝛽-HB**

**- - + + - - + + - - + +** GCM

B

anti-inflammatory genes

**- + - + - + - + - + - + 𝛽-HB**

**- - + + - - + + - - + +** GCM

*C3 Amigo2 Srgn*

A

pro-inflammatory genes

C

Rosito et al., Fig. S3

A

pro-inflammatory genes

anti-inflammatory genes

*Il1𝛽 Tnf⍺ iNos Il6 Il1⍺*

*Chil3 Arg1 Fizz1 CD206 Tgf𝛽 Il10*

*Chil3 Arg1*

*Il1𝛽 Tnf⍺ iNos*

**- + - + - + - + - + - + 𝛽-HB**

**- - + + - - + + - - + +** GCM

**- + - + - + - + 𝛽-HB**

**- - + + - - + +** GCM

B

C

D

CD

KD

Rosito et al., Fig. S4

A

Rosito et al., Fig. S5

Table 1 Diets composition and analytical components


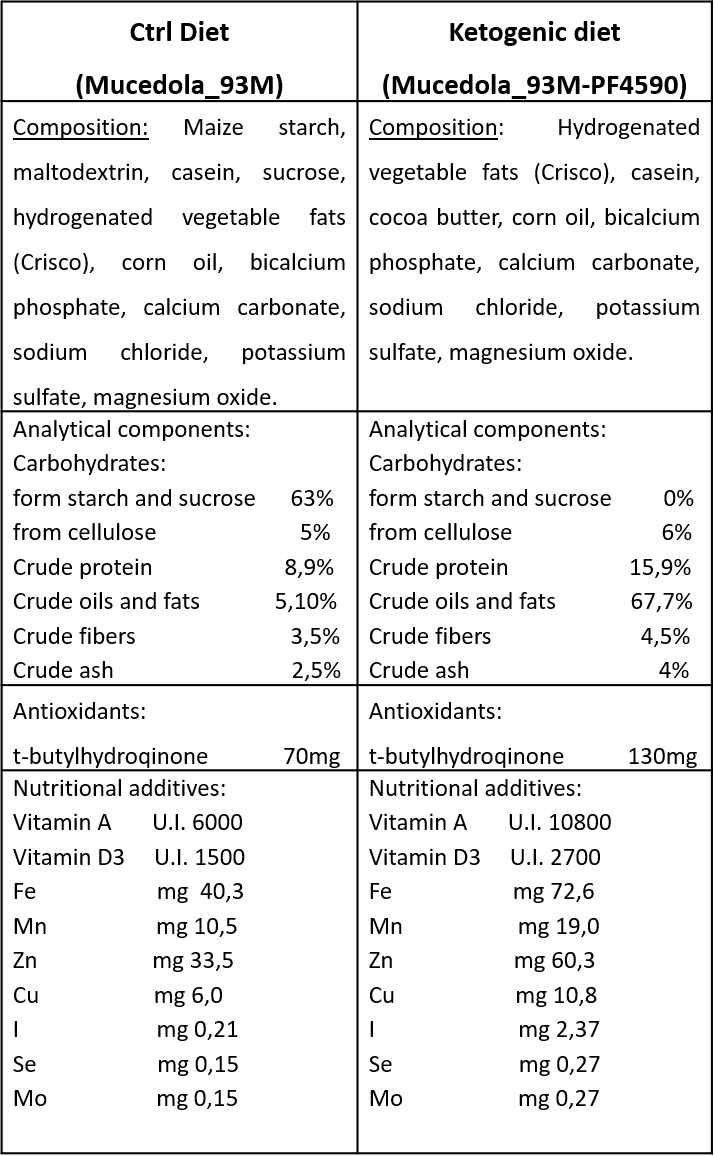

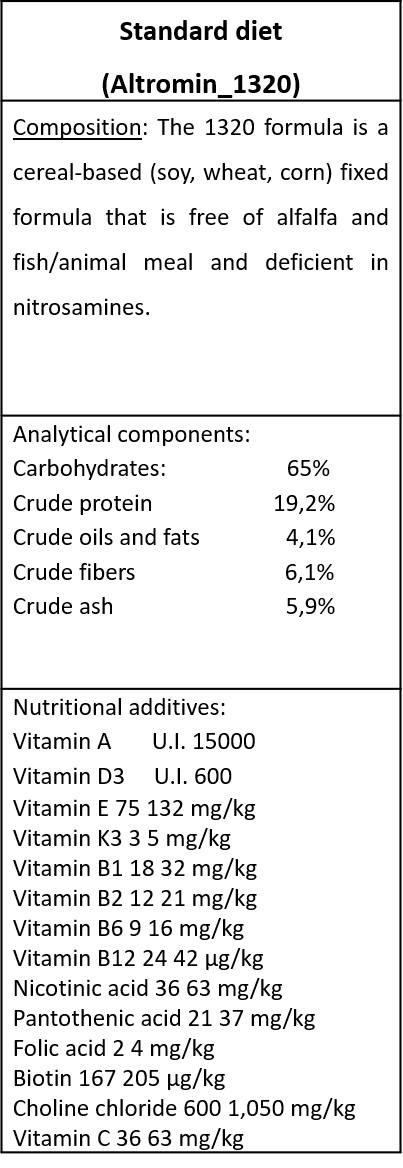


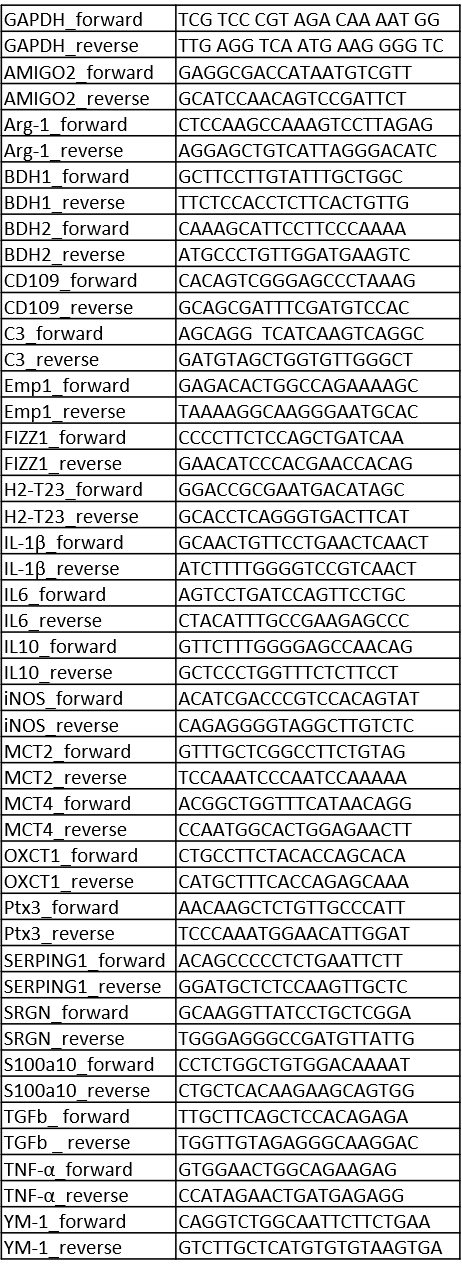


Table 2 List of oligoes
